# Physical Activity and Sleep in Adults and Older Adults in Southern Brazil

**DOI:** 10.3390/ijerph20021461

**Published:** 2023-01-13

**Authors:** Luciana Zaranza Monteiro, Joni Marcio de Farias, Tiago Rodrigues de Lima, Antônio Augusto Schäfer, Fernanda Oliveira Meller, Diego Augusto Santos Silva

**Affiliations:** 1 Physical Education Department, Federal University of Santa Catarina (UFSC), Florianópolis 88040-900, SC, Brazil; 2 Physical Education Department, Federal District University Center (UDF), Brasília 70390-045, DF, Brazil; 3 Postgraduate Program in Public Health, University of Southern Santa Catarina, Criciúma 88806-000, SC, Brazil; 4 Faculty of Health Sciences, Universidad Autónoma de Chile, Providencia 7500912, Chile

**Keywords:** physical activity, sleep quality, adults, older adult, epidemiology

## Abstract

Good sleep quality is a well-known indicator of physical and mental health, well-being, and overall vitality. This study aimed to verify the association between the practice of physical activity and sleep duration and quality in adults and older adults in southern Brazil. A cross-sectional population-based study was carried out with 820 individuals of both sexes aged 18 years or more, where sociodemographic variables were collected and also health-related variables. This study included 523 (63.8%) women and 297 (36.2%) men, and the prevalence of adequate sleep hours was 41.5% (95%CI: 39.1; 44.9). People who performed leisure walking were 34% more likely to present adequate sleep duration (PR: 1.34; 95%CI: 1.10; 1.64) compared to those who did not perform leisure walking. Individuals who met the recommendations for moderate or vigorous physical activity were more likely to have good sleep quality (PR: 1.16; 95%CI: 1.01; 1.34). Future health behavior modification strategies to improve sleep quality at the population level should consider encouraging lifestyle improvements, thus increasing the practice of physical activities.

## 1. Introduction

Good sleep quality is a well-known indicator of physical and mental health, well-being, and overall vitality [1,2]. The term “sleep quality” is widely used by researchers and involves a series of indicators such as sleep latency, number of awakenings > 5 min, wake after sleep onset, and sleep efficiency [2]. A global approach for indexing sleep quality often involves soliciting a self-rating [2]. Another term used in sleep studies is “insufficient sleep”, which is related to the number of hours/day of sleep. The Canadian guideline recommends that an adult (18 to 64 years) should have 7–9 h/day of good quality sleep, while for the older people (aged ≥ 65 years) this amount should be 7–8 h/day [3].

Sleep has long been considered a passive part of human daily lives; however, it plays a fundamental role in human life, as it has restorative, energy conservation, protective and immunological functions. In addition, sleep deprivation affects the individual’s mental and physical well-being, which leads to serious functional impairments in the performance of social roles and interpersonal relationships [1]. Some physiological mechanisms may explain the relationship between sleep and health indicators. Poor sleep quality is associated with increased levels of catecholamine, norepinephrine, and epinephrine [4]. These hormones are released into the body in response to physical or emotional stress. In addition to these hormones, the literature also shows that poor sleep quality is associated with the increased secretion of adrenocorticotropic hormone and cortisol that can cause disease [4]. 

Data from Dutch adults showed that 43.2% of them reported insufficient sleep, and 32.1% had some disorder related to inadequate sleep quality [5]. Inadequate sleep quality in Australia affected 45% of adults in 2016 [6]. A study developed in Brazil in 2014 identified that 76% of adults had at least one sleep-related problem [7]. In addition, sleep disturbances in adults have been associated with increased risk of chronic diseases including hypertension, type 2 diabetes, depression, obesity and cancer [8].

Poor sleep quality can lead to fatigue accumulation, drowsiness and mood alterations [9]. Furthermore, insufficient sleep has been negatively associated with physical performance, neurocognitive function and physical health [10]. Decreased sleep quality and duration can contribute to an imbalance in the function of the autonomic nervous system, resulting in overtraining syndrome symptoms and the elevation of inflammatory markers and, ultimately, immune system dysfunction [11]. Thus, non-pharmacological therapies, such as the practice of physical activity (PA) have been increasingly recommended, and their clinical use has been encouraged [12].

Over the past decade, studies have investigated the effects of PA on patients with sleep disorders such as sleep apnea [13]. Physical exercise has several reported effects on chronic insomnia, including improvements in sleep quality, sleep efficiency and duration, as well as decreases in sleep onset latency and wakefulness after sleep onset [12]. A recent literature review aimed to investigate the effects of physical exercise on sleep-related indicators in patients with obstructive sleep apnea, which leads to poor sleep quality [13]. Through a systematic search in different databases, the authors found nine randomized controlled trials (including 444 patients) that led to the conclusion that exercise reduces the severity of obstructive sleep apnea with no changes in body mass index, and the effect of aerobic exercise combined with resistance training is better than aerobic exercise alone in apnea–hypopnea index reduction. In addition, exercise also improves cardiopulmonary fitness, sleep quality, and excessive daytime sleepiness [13]. A recent network meta-analysis aimed to compare the effects of different intensities of acute exercise on sleep in healthy adults with good sleep [14]. The authors reported that twenty-eight studies with 325 participants met the inclusion criteria. The results revealed that there were no significant differences in terms of impact on sleep caused by different intensities of acute exercise, except when compared to no exercise [14]. For these reasons, PA is perhaps one of the most promising alternatives for improving sleep (sleep quality and number of sleep hours), because it can reduce the risk of health problems and disease through several mechanisms, including weight and inflammation reduction and increased psychological well-being [15,16,17].

Population-based studies on health behaviors are important for monitoring and guiding health promotion policies in population terms. So far, there are no policies in Brazil for monitoring the sleep indicators of the population, which limits the identification of correlates of this outcome and the definition of more specific actions for the Brazilian population. This study aimed to verify the association between the practice of PA and sleep duration and quality in adults and older adults in southern Brazil.

## 2. Materials and Methods

### 2.1. Subjects and Design 

A cross-sectional population-based study was carried out with individuals of both sexes aged 18 years or more living in the urban area of the municipality of Criciúma, Santa Catarina (SC), Brazil. Data were collected from March to December 2019 through face-to-face interviews. All information regarding the sampling and data collection process of this research has been described previously [18]. 

The study was approved by the Research Ethics Committee of the “Extremo Sul Catarinense” University under protocol number 3.084.521 on 14 December 2018, and all individuals who agreed to participate signed an informed consent form.

### 2.2. Instruments and Variables

Sleep characteristics were assessed in two ways: number of sleep hours on weekdays, and sleep quality. Questions regarding sleep hours on weekdays were: “What time do you usually go to sleep during the week (Monday to Friday)?” and “What time do you usually wake up during the week (Monday to Friday)?” From the report of sleep hours, the number of daily sleep hours was calculated, and cutoff points recently established for adequate/inadequate sleep duration for health benefits were considered [3]. For individuals aged 18–64 years, sleep duration recommendations are from seven to nine hours per night [3]. For individuals aged ≥ 65 years, seven to eight sleep hours per night is recommended [3]. From these cutoff points, according to age group, variable sleep duration (number of sleep hours) was dichotomized into “adequate” and “inadequate”.

Sleep quality was self-reported based on the following question: “How do you evaluate the quality of your sleep?”. From the answer options (very good, good, regular, poor, very poor), the variable was dichotomized into good sleep quality (very good and good categories) and poor sleep quality (regular, poor and very poor categories) [19,20].

PA was measured using the leisure and transportation sections of the International Physical Activity Questionnaire (IPAQ) (long version) [21], which consists of questions on the weekly frequency and daily duration of activities such as walking, moderate intensity PA and vigorous intensity PA. Only moderate or vigorous intensity PA (MVPA) lasting at least 10 min in a normal week were considered [22]. For the analyses, PA classifications described in Monteiro et al. [18] that are in accordance with the World Health Organization [23] were used in this study.

Variables used to describe the sample and which were considered covariates were sex (female/male), age (18–29; 20–39; 40–49; 50–59; 60–69; 70–79; ≥80 years), skin color (white, brown, black, yellow and indigenous), marital status (single, married or in stable relationship, separated or divorced, widowed), schooling (collected in successfully completed years and categorized into 0–4, 5–8, 9–11, 12 or more), paid work (yes/no), and body mass index—(BMI) (in kg/m^2^) calculated from self-reported weight and height and categorized as <25 kg/m² and ≥25 kg/m² [24].

### 2.3. Statistical Analysis

To characterize the sample, descriptive statistics were used through proportions and their respective 95% confidence intervals (95%CI). Pearson’s chi-square test was used to verify the association between dependent variables (sleep hours and sleep quality) and covariates. By dichotomizing dependent variables and verifying that they had high prevalence, for crude and adjusted analyses, Poisson regression (crude and adjusted) with robust variance was used, with *p*-value corresponding to the Wald test for heterogeneity, since analyses of cross-sectional studies with binary outcomes fit better using Poisson regression than logistic regression [25]. We chose prevalence ratios rather than odds ratios because the literature states that odds ratios can overestimate results in cross-sectional studies [25].

Regression results were presented through prevalence ratios (PR) and their respective 95%CI. In the crude analysis, associations between independent and dependent variables were individually performed. In the analysis adjusted for the model that had MVPA as independent variable, the association with the dependent variable was controlled for sex, marital status, skin color, schooling, paid work and BMI, regardless of *p*-value in the crude analysis. In the analysis adjusted for the model that had leisure walking and transportation as independent variables, the association with the dependent variable was controlled for sex, marital status, skin color, schooling, paid work, BMI and MVPA, regardless of *p*-value in the crude analysis. The significance level was set at 5%. All analyses were conducted using the Stata 13.0 statistical package (StataCorp LP, College Station, TX, USA).

## 3. Results

A total of 820 subjects were evaluated (86.1% response rate). Among them, all individuals with information for all variables (dependent, independent and covariates) analyzed were included. Table 1 details the descriptive characteristics of the sample.

**Figure 1:**
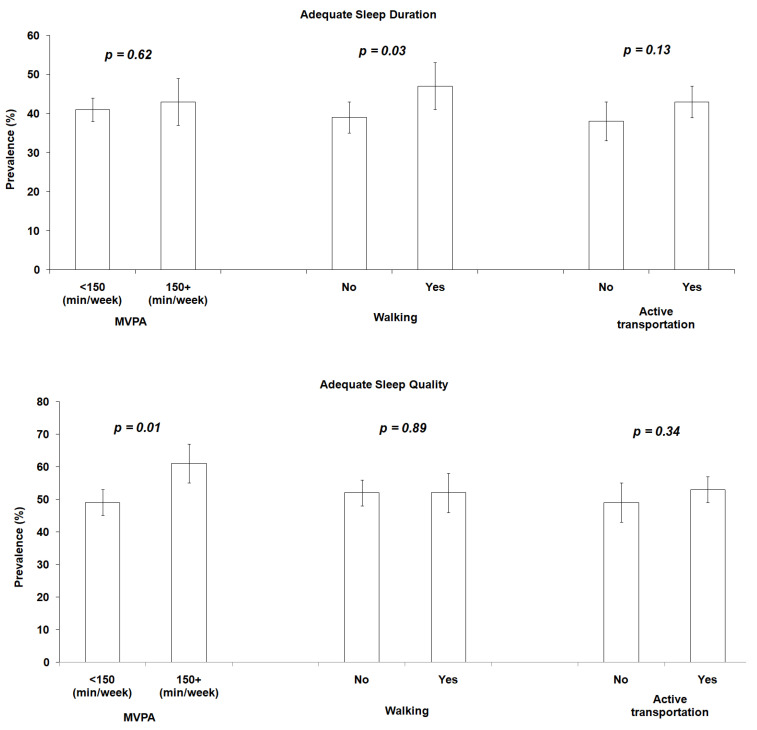
Prevalence of compliance with recommendations for moderate to vigorous physical activity, leisure walking and active transportation in individuals (≥18 years) according to adequate sleep hours and good sleep quality. Criciúma, SC, Brazil, 2019. MVPA: moderate and vigorous physical activity. Chi-square test.

MVPA recommendations were met by 25.1% (95%CI: 22.2; 28.2) of participants. The practice of leisure walking was performed by 30.0% (95%CI: 26.9; 33.2) of participants and the practice of active transportation by 65.3% (95%CI: 61.9; 68.5). Among individuals with adequate sleep duration, a higher prevalence of leisure walking (47.0%; 95%CI: 40.7; 53.2) was observed (Figure 1), while individuals with good sleep quality had a higher prevalence of compliance with MVPA recommendations (60.5%; 95%CI: 53.5; 67.0) (Figure 1).

The results for the association between MVPA, leisure walking and active transportation and adequate sleep duration and good sleep quality are shown in Table 2. In the crude analysis, leisure walking was directly associated with adequate sleep duration (PR: 1.20; 95%CI: 1.02; 1.43), whereas compliance with MVPA recommendations was directly associated with good sleep quality (PR: 1.23; 95%CI: 1.07; 1.41). Such associations remained after adjustment for possible confounding factors. Individuals who performed leisure walking were 34% more likely to have adequate sleep duration (PR: 1.34; 95%CI: 1.10; 1.64) compared to those who did not perform leisure walking. In addition, individuals who met MVPA recommendations were more likely to have good sleep quality (PR: 1.16; 95%CI: 1.01; 1.34).

## 4. Discussion

The main aim of this study was to verify the association between PA and sleep through sleep duration and quality in adults and older adults in southern Brazil. The practice of leisure walking was associated with recommended sleep duration and compliance with MVPA recommendations was associated with good sleep quality.

The mechanisms that explain the relationship between physical activity and sleep are diverse, and many authors report that there is a bidirectional relationship in these behaviors [26,27]. As the practice of physical activity is directly associated with better sleep indicators, adequate levels of sleep hours and the positive perception of good sleep quality are associated with higher levels of physical activity [27]. Changes in body temperature [28], hormone secretion [29] and heart rate [30] after exercise are some physiological mechanisms that may explain why physical activity improves sleep duration and quality, which explain the results of the present study.

Physical activity sharply increases body and skin temperature. However, before and during sleep, body temperature decreases to restore energy [28]. High-intensity exercise was associated with longer time in sleep stages three and four without rapid eye movement (NREM) [28]. More recently, moderate exercise has also been shown to lead to these adaptations [31]. Thus, it is assumed that individuals in the present study who complied with MVPA recommendations were more likely to be in NREM sleep stages three and four and, therefore reported, with greater frequency, having good sleep quality. The decrease in body temperature before and during sleep occurs to restore energy [28]. The more intense the PA, the greater the drop in body temperature during sleep and this is beneficial for energy conservation, which reduces metabolism rates and allows the body to fully relax, improving sleep quality [28,31].

The present study found that individuals who practiced leisure walking (at least 10 min) were more likely to present the recommended sleep hours when compared to those who did not. Walking is considered a type of moderate-intensity physical activity and is directly associated in previous studies with better indicators of physical and mental health in adults and older adults [32]. Systematic reviews and meta-analyses conducted in populations with and without diagnosis of diseases reported that moderate-intensity exercise is associated with adequate sleep duration [26,27]. A possible explanation for this outcome are the adaptations that the cardiovascular system undergoes with PA, such as increase in vagal modulation. The increase in vagal modulation leads to the dominance of the parasympathetic system that improves sleep indicators [30]. Furthermore, the literature suggests that the practice of walking can improve sleep quality, depressive symptoms and sleep efficiency, while decreasing night wakefulness and fatigue the next day in populations with and without diagnoses of diseases [15].

Evidence has shown that the increased practice of PA during transportation was associated with improved cardiovascular health, fewer car accidents and general reduction in healthcare costs [33,34]. In the present study, active transportation was not associated with any of the sleep indicators analyzed (duration and quality). A possible explanation for this could be the way of measuring active transportation. The questions used in this research included going to and coming from different locations (work, shopping, visiting friends, school/college) and approximately half of the investigated sample was aged 18–59 years, that is, at the age to work and/or to attend school/college. Working and/or attending school/college are considered stressors [35], especially in an economy such as that of Brazil, which has high unemployment and insecurity rates in relation to the labor market since before the COVID-19 pandemic, which results in greater self-demand for better performance [36]. These stressors lead to increased secretion of the cortisol hormone throughout the day, which reduces sleep quality [37]. Thus, it is speculated that the practice of PA during transportation has a positive effect on the health of individuals in this research; however, as the question included several scenarios recognized as stressful, individuals moved actively, but as they would go to stressful locations, the positive effects of active transportation were canceled out by the negative effects of stressors.

Some secondary results of this research are worth mentioning, such as the fact that males had a higher prevalence of good sleep quality than females. The origin of these sex differences remains unclear. A recent survey aimed to study genetic aspects to explain these differences and for that it analyzed 3544 participants from the Murcia Twin Registry [38]. The results revealed a strong genetic association between poor sleep quality and psychological distress, which accounted for 44% (95%CI: 27–61%) of the association between these two variables. Despite the remarkable sex differences in the prevalence of both poor sleep quality and psychological distress, there were no sex differences in the genetic influences on these variables. This suggests that genetic factors play a similar role for males and females in explaining individual differences in both phenotypes and their relationship [38]. Thus, as genetics has little influence on sleep quality, it is believed that environmental aspects such as lifestyle, for example, may explain these differences. As PA is an aspect of lifestyle and males practice more PA than females [32,33,34], it is suggested that PA may be a possible explanation for this difference between the sexes.

This study has several limitations that need to be mentioned. First, the bidirectional relationship between physical activity and sleep cannot be ruled out; thus, reverse causality may be present in this research, since it has a cross-sectional design. Furthermore, this design does not allow cause-and-effect relationships to be established between the investigated variables. The question about active transportation included different scenarios, which limited the identification of whether active transportation for scenarios known to be less stressful (i.e., visiting friends) would be associated with better sleep indicators. Self-reported PA measurement is also a limitation, considering that some studies have reported overestimation of these measures when compared to objective PA measurements [39]. However, for the present study, the long version of the IPAQ was used, which is widely used in population-based epidemiological surveys [39]. Another limitation of this research was the fact that the information about sleep corresponded to weekdays (not including the weekend), and the information about physical activity related to an entire week (including the weekend days). This incongruity about the questioning period of both variables can result in a lack of precision in some information.

As for the practical implications of this study, it is a fact that PA during leisure time should be encouraged as an aid strategy for adequate sleep. This fact has a direct application in public policies to promote PA in Brazil, since simple actions (such as walking, for example,) can help improve the quality of sleep in the population. Future studies should focus on different types of PA during leisure time, such as individual and collective sports practices, and check whether they are associated with sleep. These future studies may help direct PA actions at a community level.

## 5. Conclusions

In conclusion, this population-based study demonstrated the importance of PA in sleep duration and quality on weekdays. Compliance with MVPA recommendations was associated with good sleep quality on weekdays, and leisure walking was associated with recommended sleep hours on weekdays. These findings allow health professionals to guide the population towards two interchangeable behaviors, and PA promotion will result in better sleep health on weekdays.

## Figures and Tables

**Table 1:** Descriptive characteristics of the sample (≥18 years), presented in general and according to sleep duration and sleep quality. Criciúma, Brazil, 2019.

Variables	Total Sample			Adequate Sleep Duration		Good Sleep Quality
	n	% (95% CI)	n	% (95% CI)	n	% (95% CI)
**Total**	820	100	339	41.5 (39.1; 44.9)	425	51.8 (48.3; 55.2)
**Sex**				*p = 0.86*		p = 0.04 *
Male	297	36.2 (32.9; 39.6)	124	41.9 (36.3; 63.6)	168	56.5 (50.8; 62.1)
Female	523	63.8 (60.4; 67.0)	215	41.3 (37.0; 45.5)	257	49.1 (44.8; 53.4)
**Age (years)**				p < 0.01 *		*p = 0.44*
18–29	101	12.3 (10.2; 14.7)	46	45.5 (35.9; 64.0)	51	50.5 (40.6; 60.2)
30–39	93	11.3 (9.3; 13.7)	33	35.5 (26.2; 45.8)	50	53.8 (43.4; 63.7)
40–49	85	10.4 (8.4; 12.6)	47	55.3 (44.4; 65.6)	50	58.8 (47.8; 68.9)
50–59	172	21.0 (18.3; 23.9)	86	50.0 (42.5; 57.4)	78	45.4 (37.9; 52.9)
60–69	201	24.5 (21.6; 27.5)	94	47.2 (40.3; 54.2)	111	55.5 (48.2; 62.0)
70–79	129	15.7 (13.3; 18.3)	29	22.7 (16.1; 30.8)	66	51.2 (42.4; 59.7)
≥80	39	4.8 (3.4; 6.4)	04	10.3 (3.7; 25.2)	19	48.7 (32.9; 64.7)
**Marital status**				p < 0.01 *		*p = 0.22*
Single	147	17.9 (15.4; 20.7)	58	39.4 (31.8; 47.6)	82	55.8 (47.5; 63.6)
Married/stable union	495	60.4 (56.9; 63.6)	222	45.0 (40.6; 49.4)	262	52.9 (48.5; 57.3)
Separated/divorced	77	9.4 (7.5; 11.6)	33	42.8 (32.0; 54.3)	33	42.8 (32.0; 54.3)
Widowed	101	12.3 (10.2; 14.7)	26	26.0 (18.2; 35.6)	48	47.5 (37.8; 57.4)
**Skin color**				*p = 0.96*		*p = 0.87*
White	660	80.7 (77.8; 83.2)	273	41.4 (37.7; 45.3)	345	52.2 (48.4; 56.0)
Brown	91	11.1 (9.1; 13.4)	37	41.1 (31.2; 51.7)	45	49.5 (39.1; 59.7)
Black/yellow/indigenous	67	8.2 (6.4; 10.2)	29	43.3 (31.7; 55.6)	35	52.2 (40.0; 64.1)
**Schooling**				*p = 0.76*		*p = 0.34*
0–4 years	219	26.7 (23.8; 29.8)	84	38.5 (32.2; 45.2)	109	49.8 (43.1; 56.4)
5–8 years	220	26.9 (23.9; 30.0)	92	42.0 (35.5; 48.7)	111	50.5 (43.8; 57.0)
9–11 years	266	32.5 (29.4; 35.7)	114	43.0 (37.1; 49.0)	136	51.1 (45.0; 57.1)
≥12 years	114	13.9 (11.7; 16.4)	49	43.0 (34.0; 52.3)	68	59.7 (50.2; 68.3)
**Paid work**				*p = 0.83*		p < 0.01 *
No	595	72.9 (69.7; 75.8)	246	41.6 (37.6; 45.5)	291	48.9 (44.8; 55.1)
Yes	221	27.1 (24.1; 30.2)	90	40.7 (34.3; 47.3)	131	59.2 (52.6; 65.6)
**BMI**				*p = 0.71*		*p = 0.71*
<25 kg/m²	283	36.3 (33.0; 39.8)	121	42.8 (37.0; 48.6)	150	53.0 (47.1; 52.8)
≥25 kg/m²	496	63.7 (60.2; 66.9)	204	41.4 (37.0; 45.7)	256	51.6 (47.1; 56.0)

* Significant difference (Pearson’s chi-square test); MVPA: moderate to vigorous physical activity. BMI: body mass index.

**Table 2:** Crude and adjusted prevalence ratios and 95% confidence intervals of the association between physical activity and sleep variables in individuals (≥18 years) from Criciúma, SC, Brazil, 2019.

Variables	Adequate Sleep Hours				Good Sleep Quality			
	Crude Analysis		Adjusted Analysis		Crude Analysis		Adjusted Analysis	
	PR	(95%)CI	PR	(95%)CI	RP	(IC95%)	PR	(95%)CI
**MVPA ^a^**								
<150 min/week	1		1		1		1	
≥150 min/week	1.04	(0.87; 1.26)	1.03	(0.85; 1.26)	1.23	(1.07; 1.41)	1.16	(1.01; 1.34) *
**Leisure walking ^b^**								
No	1		1		1		1	
Yes	1.20	(1.02; 1.43) *	1.34	(1.10; 1.64) *	1.01	(0.87; 1.16)	0.84	(0.70; 1.01)
**Active transportation ^b^**								
No	1		1		1		1	
Yes	1.14	(0.95; 1.36)	1.11	(0.92; 1.34)	1.07	(0.92; 1.23)	1.04	(0.89; 1.21)

PR: prevalence ratio; CI: confidence interval; MVPA: physical activity of moderate and vigorous intensity. ^a^ Analysis adjusted for sex, marital status, skin color, schooling, paid work and body mass index; ^b^ Analysis adjusted for sex, marital status, skin color, schooling, paid work, MVPA and body mass index. * *p* value < 0.05.

## Data Availability

Data are available from the research coordinator and are not published. Anyone interested in the data should contact the research coordinator.
